# Respiratory Failure in an Adolescent with Primary Cardiac Sarcoma

**DOI:** 10.1155/2015/592385

**Published:** 2015-08-19

**Authors:** Daniel Angeli, Stephen J. Angeli

**Affiliations:** ^1^Department of Medicine, Montefiore Medical Center, Bronx, NY, USA; ^2^Holy Name Medical Center, USA

## Abstract

We report a case of progressive respiratory failure secondary to primary cardiac sarcoma masquerading as primary lung disease. An 18-year-old female presented to our hospital emergency department with progressive cough, dyspnea, and hemoptysis. She was treated for primary lung infection without improvement and had respiratory failure with endotracheal intubation by the third hospital day. An “intermediate” plasma brain natriuretic protein (BNP) of 216 pg/mL did not raise concerns about a heart failure diagnosis and may have delayed the correct diagnosis. Computed tomography of the chest with intravenous contrast was performed on the fifth hospital day and revealed a cardiac mass. A transthoracic echocardiogram confirmed a large left atrial mass that was obstructing mitral inflow. She was transferred to a tertiary center for emergency cardiac surgery. Primary cardiac tumors are a rare and treatable cause of heart failure in adolescent and young adult patients. Presentation can be confused with primary lung disease and must be suspected early. Plasma BNP cutoff levels used in the adult population should not be extrapolated to adolescents, as levels, both normal and abnormal, are significantly lower in this group of patients.

## 1. Introduction

An 18-year-old female with no previous medical history presented to the emergency department with a two-week history of increasing cough, fatigue, dyspnea, and hemoptysis. There was no fever. There was no recent travel. On examination, she was afebrile with respiratory rate of 20 breaths/min. and a pulse rate of 125 beats/min. Blood pressure was 104/68 mm Hg. Auscultation of her chest revealed a few scattered rales and no cardiac murmur, but a third heart sound was heard. Laboratory studies showed a white blood count of 11.0/*μ*L, neutrophils of 8.7/*μ*L (80%), and hemoglobin of 15.9 gm/dL. Oxygen saturation while breathing room air was 93%. Sputum and blood cultures were normal. Her plasma brain natriuretic protein (BNP) was 216 pg/mL and procalcitonin was <0.1 ng/mL. Chest X-ray revealed diffuse bilateral infiltrates.

Initial treatment consisted of oral azithromycin and intravenous ceftriaxone for presumed community acquired pneumonia. She failed to improve and on the second hospital day intravenous vancomycin was added to her regimen. A chest X-ray showed worsening bilateral pulmonary infiltrates and enlarging bilateral pleural effusions. By the third hospital day, her condition deteriorated further and respiratory distress resulted in endotracheal intubation and mechanical respiratory assistance. She was placed in the intensive care unit. Given the failure to improve with an antibiotic regimen, and the low procalcitonin level, the diagnosis of hypersensitivity pneumonitis was considered. Intravenous methylprednisolone was added to her regimen.

**Figure 1:**
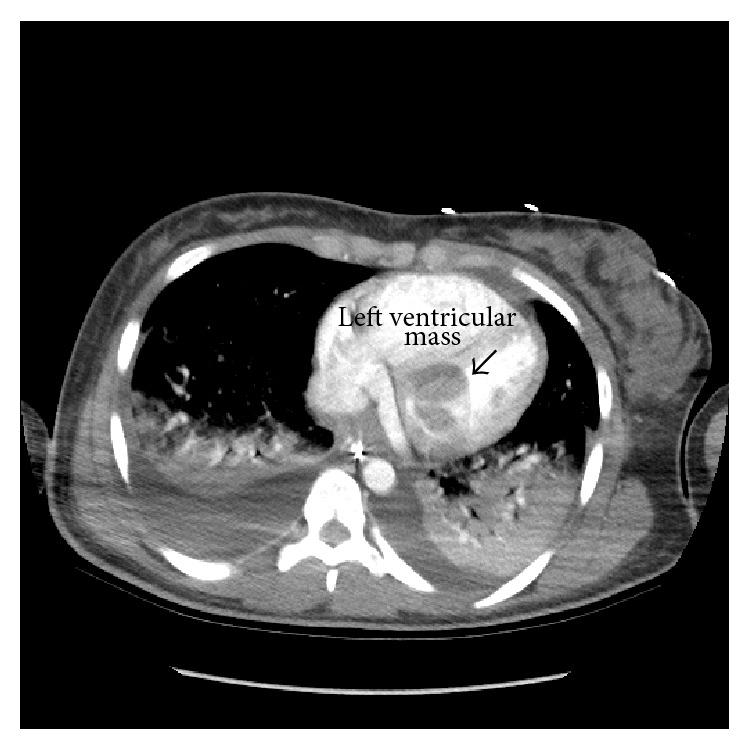
CT of the chest following intravenous contrast showing part of the mass extending into the left ventricular.

**Figure 2:**
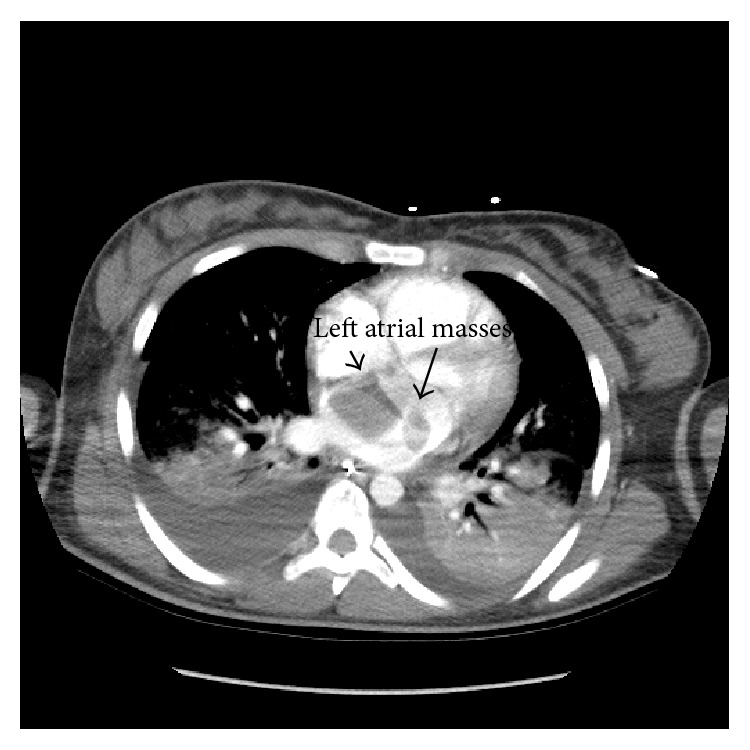
CT of the chest following intravenous contrast showing left atrial mass.

Computed tomography (CT) of the chest with intravenous contrast was performed on the fifth hospital day and it revealed “(1) bilateral pleural effusions, areas of air space consolidation involving upper and lower lobes. This is concerning for multifocal pneumonia. There is also vascular congestion. (2) Low density in the left atrium and left ventricle is nonspecific, possibly due to respiratory motion degradation. Cardiac lesions cannot be excluded. Cardiac echo is suggested” (Figures 1 and 2).

**Figure 3:**
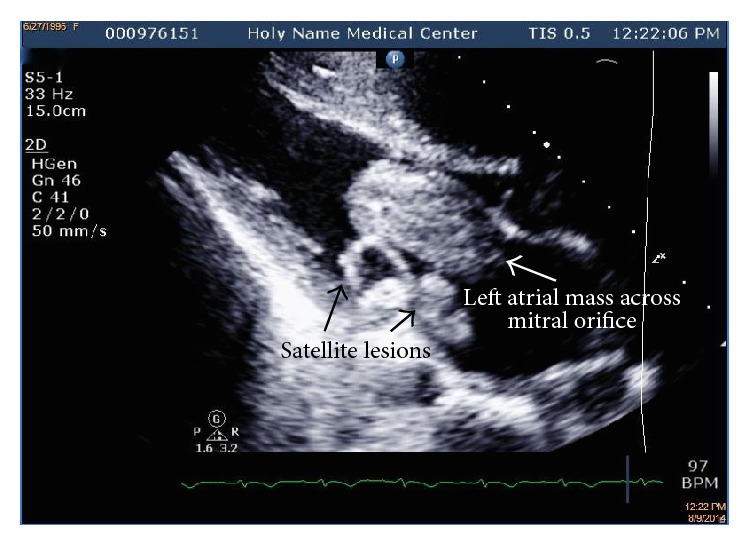
Transthoracic echocardiogram showing left atrial mass with satellite lesions and thickening of the posterior mitral leaflet.

A bedside transthoracic echocardiogram showed a large, mobile, lobulated left atrial mass with attachment to the atrial septum. The largest component of the mass measured 5.1 × 2.9 cm and prolapsed through the mitral orifice in diastole obstructing flow. Smaller satellite lesions were present and there was thickening of the posterior mitral leaflet. Left ventricular systolic function was normal. The right heart was enlarged, and the estimated right ventricular systolic pressure was estimated at 59 mm Hg (Figure 3).

**Figure 4:**
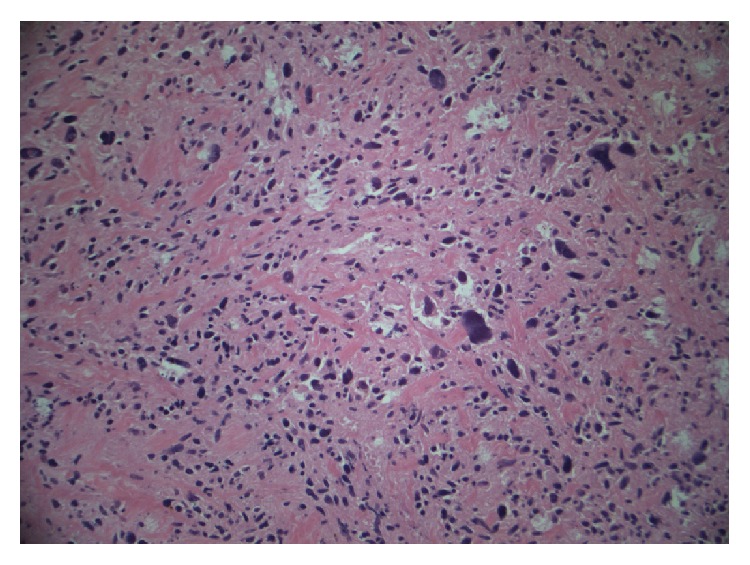
Malignant spindle cell proliferation with some very large tumor cells and areas of focal necrosis.

**Figure 5:**
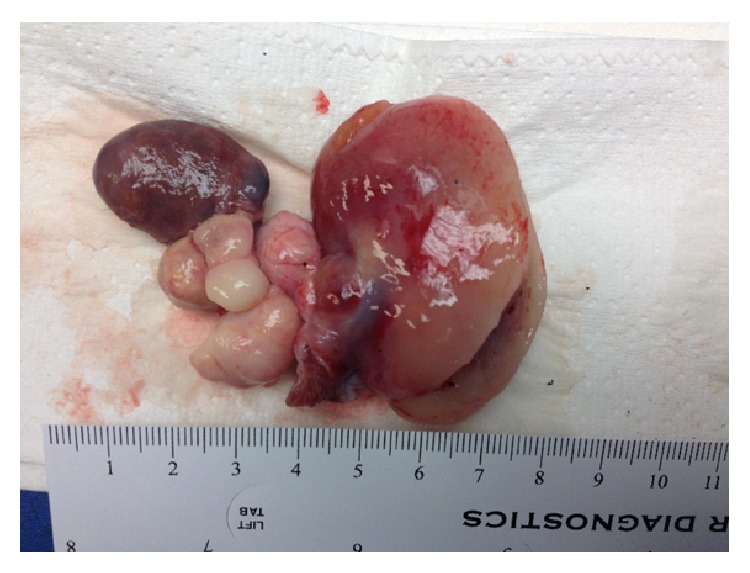
Large, multilobular mass.

The patient was referred for emergency cardiac surgery. At operation, she was found to have a large lobulated mass within the left atrium. It was attached to the septum but appeared to be growing along the free wall of the LA towards the mitral valve annulus. The valve was distorted due to the mass with thickening of the posterior leaflet. Excision of the mass and replacement of the mitral valve with a tissue prosthesis were performed. Separate lesions within the left atrium adherent to the endocardium were also sent for pathology study. Surgical pathology revealed high-grade cardiac sarcoma (malignant fibrous histiocytoma with foci of myxoid malignant fibrous histiocytoma and foci of necrosis. The tumor invades into the atrial myocardium and involves the lateral margins of the atrial tissue present in the specimen) (Figures 4 and 5).

Her postoperative course was generally satisfactory. She has since been treated with chemotherapy.

## 2. Discussion

This is the case of a critically ill 18-year-old female who was diagnosed with a malignant sarcoma arising in the left atrium. She rapidly deteriorated over several days with life-threatening respiratory failure and presented a clinical puzzle to the clinicians involved in her care. Fortunately, the correct diagnosis was finally made and the patient was promptly treated. Our patient was admitted to the adult medicine service. In addition to a discussion on the features of this rare and dangerous condition, we will also discuss how adolescents and children can differ from adults in certain key measures and how an appreciation of this may have led to an earlier diagnosis.

Primary tumors of the heart are extremely rare. Based upon the data of 22 large autopsy series, the frequency of primary cardiac tumors is approximately 0.02%, corresponding to 200 tumors in 1 million autopsies. Metastatic tumors to the heart are far more common [1]. In adults, myxomas are the most common benign tumors and in children, rhabdomyomas and fibromas are the most common. Of primary cardiac tumors, malignant tumors constitute only 17% [2].

Cardiac tumors may produce symptoms based on their location in the heart and whether they interfere with normal cardiac function. Some cardiac tumors are found incidentally. Left atrial and left ventricular tumors can present with systemic complaints of fever, chills, and fatigue, as well as symptoms specific to the hemodynamic effects of mitral obstruction, as was the case with our patient. Other potential presenting findings may be systemic embolization, arrhythmias, chest pain, syncope, and presyncope [3].

Our patient presented with respiratory symptoms that were initially treated as community acquired pneumonia and then, when she failed to improve, as a case of environmentally mediated hypersensitivity pneumonitis. Severe pulmonary edema secondary to the presence of an obstructing left heart mass with interference of normal mitral function was later documented. The observed brain natriuretic peptide (BNP) level of 216 pg/mL is an “indeterminate” level for the adult population in which this biomarker was first studied. The young age of our patient very likely affected the magnitude of the BNP level and deserves closer examination.

BNP is a 32-amino-acid polypeptide secreted by the ventricles of the heart in response to excessive stretching of myocardial cells. BNP has been studied extensively and is now in widespread clinical use as an aid in the diagnosis of patients with dyspnea. In the adult population, dyspneic patients with heart failure have BNP values of >400 pg/mL. Values <100 pg/mL have a good negative predictive value and heart failure can be excluded. Values between 100 and 400 pg/mL are considered “gray zone” elevation and can be seen in a variety of clinical settings, and clinical judgment is generally recommended [4].

Plasma BNP levels have been shown to have wide individual variability. Such factors as age, sex, renal function, and type of heart failure can affect levels. Normal BNP is known to increase with increasing age [5]. In a multicenter study of 161 children and adolescents with symptomatic systolic heart failure (class II–IV), studying the effects of high versus low dose carvedilol, the median initial plasma BNP across all groups was 110 pg/mL [6]. In another study of 163 children and adolescents with moderately symptomatic class II and III heart failure, the median BNP was 110 pg/mL, compared with 20 to 40 pg/mL reported in normal children. A BNP level ≥140 pg/mL in this cohort was predictive of adverse outcomes [7]. It is therefore not advisable to extrapolate the BNP cutoff values obtained in the adult population to children and adolescents. Seen in this light, it is possible that the observed BNP in this case may have raised the possibility of heart failure earlier in her course.

Depending on the location and type of tumor, surgery may or may not be necessary. Because of the risk of embolization or hemodynamic compromise, left side lesions, such as myxomas, should undergo resection. In the case of malignant cardiac tumors, surgical excision in combination with systemic chemotherapy remains the best available treatment [8].

To make the diagnosis, imaging studies are essential and will be presented briefly. Certain features of the mass are more suggestive of malignancy. These include broad-based mass, invasion of surrounding tissue, mass presence in more than a single chamber, poor definition of mass border, tissue inhomogeneity, large size >5 cm, and presence of either pericardial or pleural effusion [9].

Two-dimensional echocardiography is often the initial diagnostic modality of choice. It is readily available and has very good sensitivity of 93%. Transesophageal echocardiography is also excellent with a sensitivity of 97% [10]. In addition to morphology, echocardiography provides hemodynamic information on regurgitation and stenosis as well as estimation of right ventricular pressure. Limitations to echocardiography include limited field of view and modest ability to differentiate tumor types and interference from lung and other noncardiac tissue. Tissue characterization can be enhanced by the use of echocardiographic contrast perfusion imaging. Such modality would better differentiate the neovascularization of malignancies from the avascularity of thrombi and the sparse vascularity of stromal tumors, such as myxomas [11]. Cardiac magnetic resonance (CMR) has become the imaging modality of choice in its ability to provide high spatial resolution, wide view of the heart and surrounding structures and give accurate tissue characterization. While CMR criteria for distinguishing benign from malignant tumors are highly accurate, histologic diagnosis still remains the “gold standard.” Computed tomography (CT) with electrocardiographic gating is another tool that can provide important structural information in the assessment of cardiac masses [12].

## 3. Conclusion

Cardiac tumors are a rare and treatable cause of heart failure in adolescent and young adult patients. Only timely diagnosis and treatment will give the patient the best chance at survival. The presentation may be confused with primary pulmonary disease and must be suspected early. An array of cardiac imaging studies, each with their respective strengths, can be used to establish the diagnosis. Caution must be employed when interpreting plasma BNP levels, taking into account the significantly lower levels seen in young patients.
